# Transcriptomic Profiling of Monozygotic Twins with Type 1 Gaucher Disease

**DOI:** 10.3390/life16050741

**Published:** 2026-04-29

**Authors:** Aslı İnci, Sümeyye Aydoğdu Demirel, Filiz Başak Cengiz Ergin, Gürsel Biberoğlu, İlyas Okur, Fatih Süheyl Ezgü, Leyla Tümer, Rıdvan Murat Öktem, Serap Dökmeci

**Affiliations:** 1 Department of Pediatric Nutrition and Metabolism, School of Medicine, Gazi University, Ankara 06500, Türkiye; 2 Department of Medical Biology, School of Medicine, Hacettepe University, Ankara 06100, Türkiye

**Keywords:** Gaucher disease, twins, RNA sequencing, qRT-PCR

## Abstract

**Background**: Gaucher disease (GD) arises from pathogenic variants in the *GBA1* gene and is known for its wide range of clinical presentations—a variability that genotype alone cannot adequately account for. **Objective**: This study aimed to explore transcriptomic factors that might help explain why two genetically identical twins with type 1 GD developed noticeably different clinical outcomes. **Methods**: We isolated peripheral blood mononuclear cells from both twins and two age-matched controls, then differentiated them into macrophages in vitro before conducting RNA sequencing. Gene expression differences were analyzed using established bioinformatics pipelines, and a subset of genes were subsequently assessed by quantitative real-time PCR (qRT-PCR) to confirm the sequencing findings. **Results**: Both twins shared a GD-associated transcriptional signature broadly reflecting immune activation and lysosomal stress. Interestingly, the twin who experienced systemic complications had a relative enrichment of interferon-responsive transcripts, while the less severely affected twin showed more pronounced suppression of small nucleolar RNA clusters. That said, neither difference held up after correcting for multiple comparisons, so these patterns are best viewed as exploratory trends rather than definitive findings. The qRT-PCR results lend partial support to this picture: stress- and immune-related genes (*DDIT4*, *RPH3A*, *SAMSN1*) trended toward higher expression in patients versus controls, and interferon-stimulated genes (*ISG15*, *RSAD2*, *IFI44L*) were more elevated in M2 than in M1. **Conclusions**: Taken together, these findings suggest that factors beyond genetics—whether epigenetic, environmental, or otherwise—may play a meaningful role in shaping how GD manifests differently even between individuals with identical DNA. Although the data are preliminary, they point to transcriptomic profiling, paired with targeted validation, as a useful starting point for building hypotheses about why this disease looks so different from one patient to the next, even when the underlying mutation is the same.

## 1. Introduction

Gaucher disease (GD, OMIM #230800, ORPHA355) is the most frequent lysosomal storage disorder within the group of sphingolipidoses. GD is an autosomal recessive disorder resulting from pathogenic variants in the *GBA1* gene, located on chromosome 1q21. These mutations cause a significant reduction in the activity of the lysosomal enzyme glucocerebrosidase (GCase; also referred to as glucosylceramidase or acid β-glucosidase). Normally, this enzyme catalyzes the breakdown of glucosylceramide into ceramide and glucose [1].

Gaucher disease represents an inherited lysosomal storage disorder that primarily involves cells of the mononuclear phagocyte system. The condition arises from defective degradation of glucocerebroside, a lipid normally derived from the breakdown of membrane components during blood cell turnover. As a result, macrophages progressively accumulate this substrate, developing into the characteristic lipid-laden “Gaucher cells”.

The clinical spectrum is heterogeneous, but three main subtypes are traditionally recognized: type 1, the most prevalent, is usually non-neuronopathic, represents the majority of cases; types 2 and 3 are associated with neurological manifestations. Type 2 corresponds to the acute neuronopathic variant, characterized by rapidly progressive neurological decline in infancy, hydrops fetalis, seizures, ichthyosis [2], typically leading to death within the first two years of life. Type 3, often referred to as the chronic neuronopathic form, includes individuals who survive beyond infancy but present with neurological involvement of varying severity. In some patients, this may be limited to impaired horizontal saccadic eye movements, while others develop more pronounced features such as progressive neurodegeneration, myoclonic epilepsy, or psychiatric disturbance [1,2,3].

Clinically, over 90% of patients with GD1 present with splenomegaly, sometimes massive, and often accompanied by thrombocytopenia, anemia, or hyperferritinemia; hepatomegaly is also common, though progression to fibrosis or cirrhosis is rare [4]. Hemorrhagic manifestations such as epistaxis or perioperative bleeding are usually linked to thrombocytopenia and platelet dysfunction, while anemia is moderate and leukopenia uncommon [1]. Skeletal involvement ranges from asymptomatic imaging findings to chronic pain, osteonecrosis, and acute bone crises, particularly in untreated or splenectomized individuals [5,6]. Women with severe disease may experience symptom exacerbation during pregnancy, especially with postpartum hemorrhage, though fertility is generally preserved [7].

The relationship between genotype and clinical presentation in Gaucher disease remains complex, with marked variability that is not yet fully understood [8,9]. Nonetheless, certain patterns have been recognized; for instance, the p.Asn409Ser variant is consistently linked to type 1 disease and is considered protective against neuronopathic involvement. Despite these associations, clinical outcomes can differ substantially, and cases of discordant phenotypes have even been observed in individuals with identical genetic backgrounds, including monozygotic twins [10,11].

Nevertheless, limited information is available regarding gene expression patterns and their impact on different molecular pathways in monozygotic twins in Gaucher disease. Based on this rationale, this study investigated the gene expression profiles of adult patients who were monozygotic twins diagnosed with type 1 GD.

## 2. Materials and Methods

This exploratory, prospective study received ethical approval on 12 September 2025. Experimental procedures were initiated three days later following immediate study preparation. The workflow included monocyte isolation and in vitro macrophage differentiation (5 days), followed by RNA extraction, library preparation, RNA sequencing, and quantitative real-time polymerase chain reaction (qRT-PCR) analysis. This investigation included blood samples from two adult twins diagnosed with type 1 GD and 2 healthy controls. The study included two monozygotic twins (M1 and M2), both 43-year-old females. They were diagnosed with GD after having anemia, bleeding problems (epistaxis, long and abundant menstrual bleeding) due to thrombocytopenia, and having a distended abdomen due to hepatosplenomegaly confirmed with low levels of enzyme. The enzymatic assays revealed markedly reduced GCase activity in both patients (in M1 0.25 nmol/h/ mg protein and in M2 0.21 nmol/h/ mg protein (normal: 9.4 ± 3.2)). The *GBA* gene mutation was homozygous *GBA* mutation confirming type 1 GD (p.Asn409Ser/p.Asn409Ser) (Table 1 shows the demographic characteristics of patients and healthy individuals). Despite sharing identical genetic mutations, M1 and M2 demonstrated distinct clinical trajectories and organ involvement. M1 presented with systemic complications that were absent or attenuated in M2. Hypertension was documented in M1 but not in M2. Furthermore, M1 developed an anaphylactic reaction to enzyme replacement therapy (ERT), while M2 tolerated therapy without hypersensitivity. A striking contrast was observed in renal function. M1 demonstrated nephrotic-range proteinuria (4401 mg/day), while M2 exhibited only mild proteinuria (206 mg/day). This disparity strongly indicates differential renal involvement despite genetic concordance. Cardiovascular evaluation by echocardiography revealed mild diastolic septal and wall thickening in M1 (1.2 cm and 1.1 cm, respectively), whereas M2’s cardiac measurements were within normal limits (0.9 cm and 0.8 cm). Abdominal ultrasonography and magnetic resonance imaging (MRI) identified hepatomegaly and splenomegaly in both twins. Neurological assessment with electroencephalography revealed mild diffuse cerebral dysfunction in both twins without any symptoms detected while screening with both cranial MRI and electroencephalography (Table 2).

The control cohort consisted of two age matched healthy individuals (C1: 43-years-old male; and C2: 45-year-old female) without *GBA* mutations. The enzyme levels and gene mutations were normal (C1: 8.0; C2: 10.3 nmol/h/mg protein).

Peripheral blood mononuclear cells (PBMCs) were isolated from 24 mL of peripheral blood by density gradient centrifugation using Ficoll–Paque PLUS (GE Healthcare, Chicago, IL, USA). Monocytes were subsequently differentiated into macrophages by treatment with M-CSF (10 ng/mL; Sigma-Aldrich, Burlington, MA, USA) in RPMI 1640 supplemented with 10% FCS (Gibco, Waltham, MA, USA) for 5 days. Macrophage maturation was confirmed by light microscopy and flow cytometry using CD14 and CD68 antibodies (eBioscience, San Diego, CA, USA) [12,13].

### 2.1. RNA Isolation, Library Preparation, and Sequencing

Total RNA was extracted from macrophages using the TRIzol method, libraries were prepared with the Illumina Total RNA Prep Kit (Illumina, USA, #20040529), which includes rRNA depletion, RNA fragmentation, cDNA synthesis, adapter ligation, and purification, following the manufacturer’s protocol, and sequencing was performed on the Illumina NovaSeq 6000 (Illumina, Inc., San Diego, CA, USA) (average 30 M paired-end 150 bp reads per sample) according to the manufacturer’s instructions [14]. Detailed procedures are provided in the Appendix A.

### 2.2. Bioinformatics Analysis

Bioinformatics analysis was performed using FASTQC, Trimmomatic v0.39 [15], the Illumina DRAGEN RNA Pipeline with GENCODE v40, and differential expression/functional enrichment tools including edgeR [16], TopGO [17], and clusterProfiler [18]; details are provided in the Appendix A.

### 2.3. cDNA Synthesis and Quantitative Real-Time PCR (qPCR)

For cDNA synthesis, 1 µg of total RNA was reverse-transcribed using the iScript cDNA Synthesis Kit following the manufacturer’s instructions.

Gene expression levels were quantified by qRT-PCR on a Roche LightCycler 480 system using SYBR Green Master Mix. Primers were designed to target *ISG15*, *RSAD2*, *IFI44L*, *DDIT4*, *RPH3A*, and *SAMSN1*; sequences are listed in Table 3.

All reactions were performed in triplicate, and melting curve analysis confirmed a single specific amplification product for each primer pair. Primer melting temperatures ranged from 60.2 °C to 62.9 °C across the six primers used; while this range is slightly wider than ideal, all primers were tested under a uniform annealing temperature of 60.5 °C and produced clean, single-peak dissociation curves, supporting their specificity under the conditions used. Amplification efficiencies were within the acceptable range (80–110%) for all assays.

*GAPDH* was used as the reference gene for normalization. To verify its suitability, we confirmed that *GAPDH* Ct values were stable across all four samples, with an inter-sample variation of less than 0.5 cycles, indicating consistent expression under the experimental conditions applied.

## 3. Results

### 3.1. Global Transcriptomic Landscape

**Figure 1:**
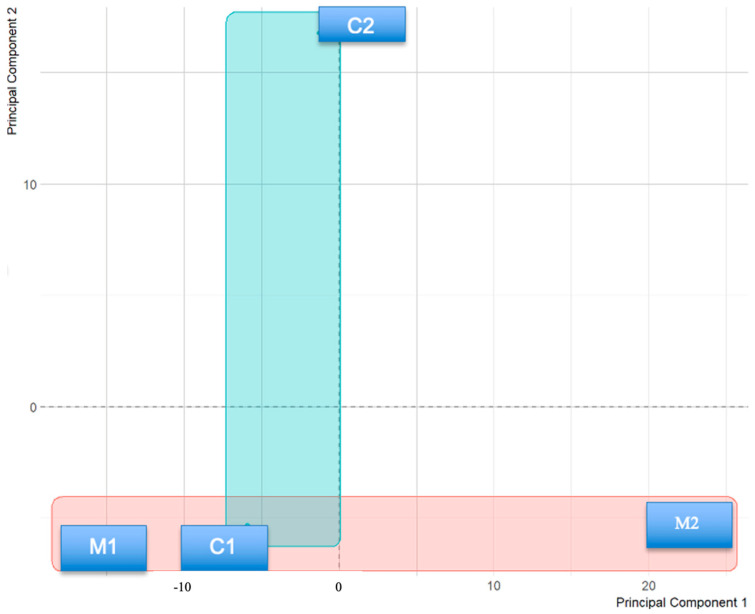
The distribution of samples on the PC1 and PC2 plane after PCA (Principal Component Analysis of Abundant Genes).

As an initial step, we applied principal component analysis (PCA) to the RNA sequencing data from patient (M1, M2) and control (C1, C2) macrophages. Patient samples tended to occupy a distinct region of the PCA plot relative to controls along the first principal component, pointing to differences in the overall transcriptional landscape between the two groups (Figure 1). Given that only two patients and two controls were included, we are cautious about overinterpreting this separation; nonetheless, it suggests that GD macrophages may differ from healthy macrophages in ways that extend beyond any single gene.

**Figure 2:**
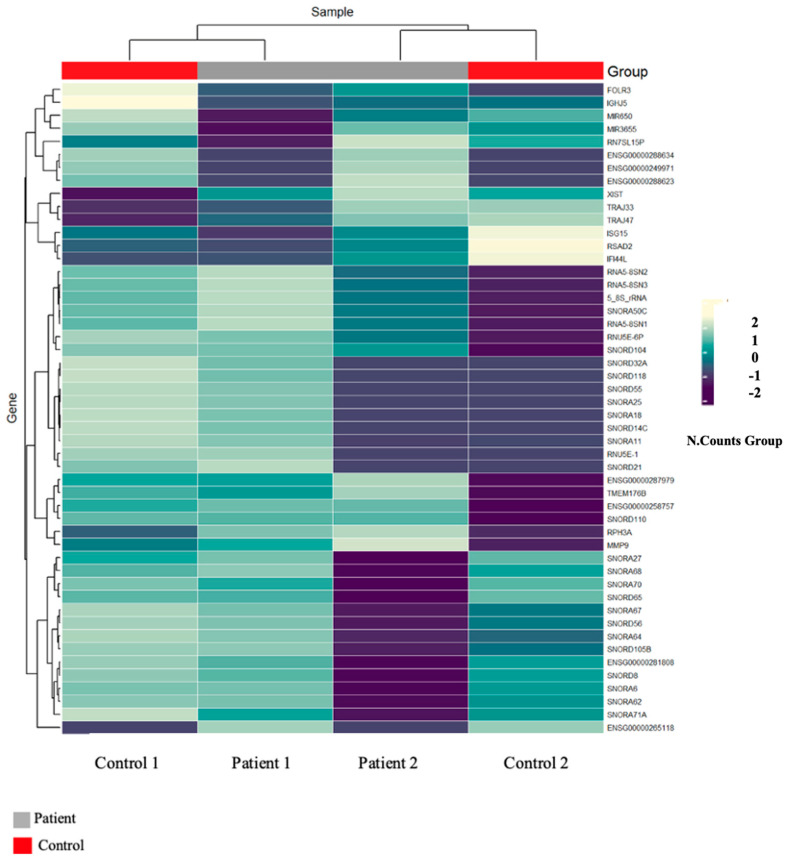
Expression profiles of the top 50 genes showing the highest variation among samples.

We also examined the 50 most variably expressed genes through unsupervised hierarchical clustering, which produced a heatmap in which patient and control samples fell into separate groups (Figure 2). None of the individual expression differences survived FDR correction—an outcome we attribute primarily to the small sample size rather than a true absence of biological signal. On balance, the PCA and clustering data raise the possibility that GD patient macrophages carry a shifted transcriptional profile, though this remains tentative and will need to be tested in larger cohorts.

### 3.2. Shared Transcriptional Signature of Monozygotic Twins

Looking at the twins individually, M1 and M2 grouped closely together on the heatmap and fell apart from the control samples (Figure 2). The fact that both patients clustered together—rather than one sitting closer to controls—hints that they share a transcriptional background tied to their disease, which is perhaps unsurprising given their identical genetic makeup and common diagnosis.

**Figure 3:**
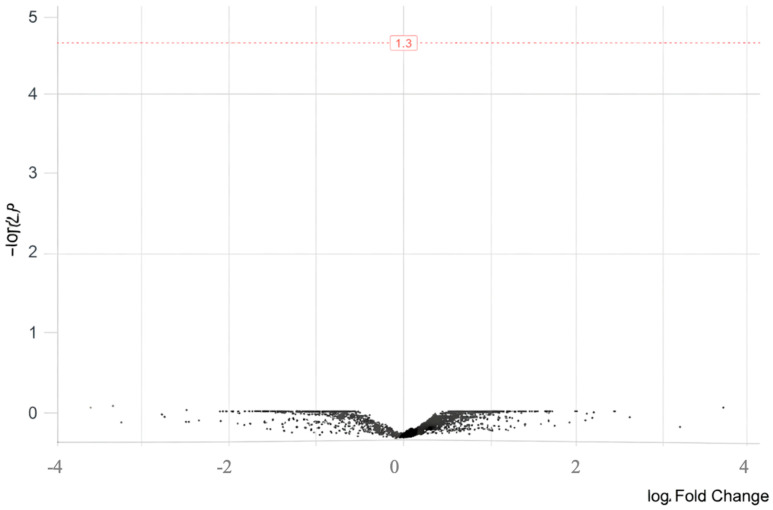
A volcano plot of the genes showing the most significant variability. No gene passed the FDR correction. The horizontal red dashed line represents the statistical significance threshold of (corresponding to a *p*-value of 1.3). Points above this line indicate significantly differentially expressed genes/transcripts.

We next carried out differential expression analysis to identify genes with altered expression between patients and controls after FDR adjustment. Several genes showed variability that caught our attention, yet none crossed the threshold (Table 4). The volcano plot gave a broad picture of how expression was distributed across the genome and helped visualize the directional tendencies we describe below, even though no individual gene stood out as a significant hit with FDR adjustment (Figure 3).

On the upregulation side, three genes tied to stress signaling and immune activation—*RPH3A*, *SAMSN1*, *and DDIT4*—trended higher in both M1 and M2 relative to controls, with no significant FDR adjustment.

### 3.3. Transcriptional Divergence Between M1 and M2

While M1 and M2 shared the broad transcriptional shifts described above, a closer look at normalized expression intensities revealed some differences between the two. M1 stood out for somewhat higher expression of transcripts linked to cellular stress and RNA processing—among them *SAMSN1*, *DDIT4*, and a handful of small nucleolar RNAs (snoRNA) and spliceosome-associated genes. The drop in ribosomal pseudogene expression also looked more pronounced in M1 than in M2, though the magnitude of this difference was mild with no significance in FDR.

Where the twins diverged most noticeably was in their interferon-related transcripts. *ISG15*, *RSAD2*, *IFI44L*, *MIR650*, and *MIR3655* all ran modestly lower in M1 and higher in M2. These genes feed into interferon signaling and innate immune regulation, so their opposite pattern in the two twins is intriguing without significance.

M2 showed a somewhat quieter picture in the ribosomal and RNA-processing space. A group of small nucleolar RNAs—*SNORD32A*, *SNORD21*, *SNORA11*, *SNORA18*, *SNORA25*, and *RNU5E1*—were modestly reduced in M2 without significance in FDR adjustment.

### 3.4. qRT-PCR Validation of Stress- and Immune-Related Genes

**Figure 4:**
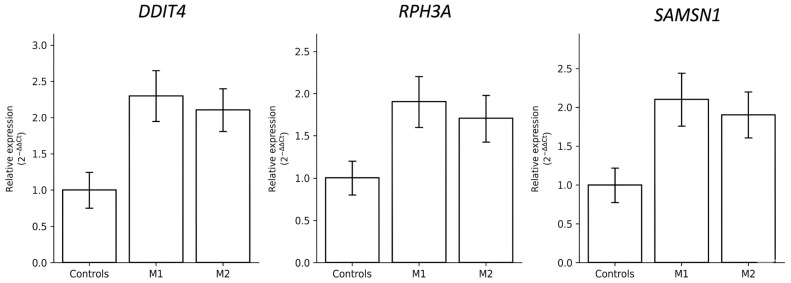
qRT-PCR validation of *DDIT4*, *RPH3A*, and *SAMSN1* expression. Relative expression levels (2^−ΔΔCt^) were higher in patients than in controls. Mean fold changes were 2.3 (*DDIT4*), 1.9 (*RPH3A*), and 2.1 (*SAMSN1*) relative to controls. Relative expression levels are presented as 2^−ΔΔCt^ values normalized to the housekeeping gene. No inferential statistics are reported; with *n* = 2 biological samples per group, valid significance testing is not feasible.

To check whether the RNA sequencing trends held up under an independent method, we ran qRT-PCR for *RPH3A*, *SAMSN1*, and *DDIT4* in both patients and controls. All three target genes came back higher in patients than in controls. Mean expression relative to controls was 2.30-fold for *DDIT4*, 1.90-fold for *RPH3A*, and 2.10-fold for *SAMSN1* in M1, and 2.10-fold, 1.70-fold, and 1.90-fold, respectively, in M2 (Figure 4).

The primary value of this validation exercise lies elsewhere: the directionality of the qRT-PCR results was concordant with the RNA sequencing data for all three genes. Every gene that trended upward in the sequencing analysis also came back elevated by qRT-PCR, across both patients and independently of the platform used. This cross-method directional consistency strengthens the case that the observed upregulation is unlikely to be a technical artifact of the sequencing workflow, even though it falls short of statistical confirmation.

Between the twins, M1 showed somewhat higher expression than M2 for all three genes, echoing the pattern seen in the heatmap. Given the sample size, we note this only as a directional tendency.

### 3.5. qRT-PCR Assessment of Interferon-Stimulated Genes

**Figure 5:**
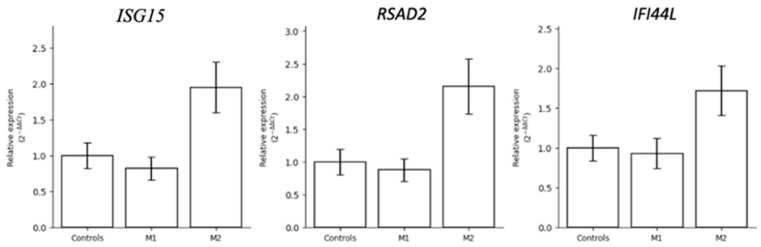
qRT-PCR validation of interferon-stimulated gene expression. Relative expression levels (2^−ΔΔCt^) of *ISG15*, *RSAD2*, and *IFI44L* were higher in M2 compared with M1 and controls. Mean fold changes relative to controls were 1.95 (*ISG15*), 2.15 (*RSAD2*), and 1.72 (*IFI44L*). Relative expression levels are presented as 2^−ΔΔCt^ values normalized to the housekeeping gene. No inferential statistics are reported; with *n* = 2 biological samples per group, valid significance testing is not feasible.

To follow up on the inter-twin differences in interferon-related transcription suggested by the RNA sequencing data, we measured *ISG15*, *RSAD2*, and *IFI44L* expression by qRT-PCR in both twins and controls. All three interferon-stimulated genes came back higher in M2 than in both M1 and controls, which lines up with what the sequencing data had suggested. M1, by contrast, sat closer to—or in some cases below—control levels for these transcripts. Mean expression relative to controls was 1.95-fold for *ISG15*, 2.15-fold for *RSAD2*, and 1.72-fold for *IFI44L* in M2, whereas M1 remained close to or below control levels (*ISG15*: 0.82-fold, *RSAD2*: 0.88-fold, *IFI44L*: 0.93-fold) (Figure 5). The sample size does not permit valid inferential statistics, and we present these results as descriptive fold changes only.

The directional agreement between qRT-PCR and RNA sequencing for all three genes—M2 higher, M1 lower—is noted, though we recognize that this cannot be taken as statistical confirmation. With only two biological samples per group, no valid significance test is available, and we present this observation purely as a descriptive finding. The consistency across two independent methods is offered only as a basis for generating hypotheses in future work with larger cohorts, not as evidence of a confirmed biological difference.

## 4. Discussion

The question driving this study was one we could not answer with a conventional cohort design: if two people share the same *GBA1* mutation, the same genetic background, and have grown up in broadly comparable circumstances, what accounts for the fact that one develops more severe disease than the other? Monozygotic twins give us a rare opportunity to hold the genotype constant and look for answers elsewhere—in the transcriptome, in the epigenetic state, or in the environmental history. The data we present here are preliminary, and we want to be direct about that from the outset. No gene survived FDR correction. The sample group included two patients and two controls. Everything that follows is hypothesis-generating, and we have tried to describe it as such throughout.

The broader question of why two individuals sharing identical *GBA1* mutations follow such divergent disease trajectories remains unresolved. One consideration that deserves direct acknowledgment is the differential immunological history of the two twins, despite their otherwise comparable ERT exposure. Both twins were initially treated with imiglucerase, during which M1 developed an anaphylactic reaction and subsequently required a formal desensitization protocol. This protocol necessitated corticosteroid, antihistamine, and paracetamol premedication prior to each infusion every two week for 4 months. Later on, the patient only used paracetamol and antihistaminics and desensitization protocol for imiglucerase infusion. Both twins were later transitioned to taliglucerase alfa, under which neither twin required premedication; this switch occurred approximately eight years before the time of sampling. The twins received taliglucerase alfa on a biweekly schedule for approximately seven years (7.2 years) until supply-related issues prompted a further change in therapy. Both twins have since been receiving velaglucerase alfa for the past eight months without any hypersensitivity reactions. During the initial three months of velaglucerase alfa therapy, M1 received infusions over an extended duration with paracetamol and diphenhydramine premedication only, without corticosteroid. Over the subsequent five months, infusions were administered at the standard biweekly schedule and at the routine infusion rate, with no premedication required. At the time of sampling the patients had been receiving velaglucerase alfa with the same protocol. Although the period of corticosteroid exposure is therefore temporally remote, we cannot fully exclude the possibility that repeated steroid premedication during the imiglucerase desensitization phase left lasting immunological imprints on M1’s macrophage transcriptome—a confounding factor that transcriptomic data alone cannot resolve.

Given that ERT has been shown to reshape macrophage gene expression profiles [19,20], this differential exposure history represents a plausible—and arguably more parsimonious—explanation for at least part of the inter-twin transcriptomic divergence we observed.

The PCA and hierarchical clustering results both pointed in the same direction: M1 and M2 sat together in transcriptional space and apart from the controls, while the 50 most variably expressed genes produced a heatmap in which patient and control samples fell into distinct groups. We were cautious about this. With a total number of four individuals, a clean PCA separation is not surprising on statistical grounds alone, and we would not want to overread it. What we do think is worth noting is that both twins clustered together rather than one sitting closer to controls—which at least suggests that whatever is driving the separation is tied to their shared disease state, not to something individual-specific. That observation is consistent with prior work showing that *GBA1* deficiency produces a broadly altered macrophage phenotype, driven by glucosylceramide accumulation and downstream lysosomal dysfunction [21,22].

Among the genes trending upward in both M1 and M2 relative to controls, *DDIT4*, *RPH3A*, and *SAMSN1* were the most consistent, and all three were subsequently assessed by qRT-PCR. Mean expression relative to controls was 2.30-fold for *DDIT4*, 1.90-fold for *RPH3A*, and 2.10-fold for *SAMSN1* in M1, and 2.10-fold, 1.70-fold, and 1.90-fold, respectively, in M2. What they offer is directional concordance across two independent methods—RNA sequencing and qRT-PCR—for all three genes, which makes a purely technical artifact less likely and provides a basis for hypothesis generation in future studies with larger cohorts.

*DDIT4* is a stress-responsive inhibitor of mTORC1. Kim et al. [23] reviewed *DDIT4*’s pathophysiological functions in detail, describing it as a stress-induced protein that controls metabolism, oxidative stress, autophagy, and cell fate, all processes directly relevant to a disease defined by lysosomal substrate accumulation [21,24]. Sunilkumar and Dennis [25] argued that chronically elevated *DDIT4* contributes to the pathogenesis of metabolic complications partly through sustained Akt/mTORC1 suppression, and that this makes it a candidate therapeutic target in metabolic disease more broadly. The *DDIT4* elevation we observed in both twins is consistent with a picture of chronic mTOR suppression in GD macrophages, a finding that lines up with prior reports of dysregulated autophagy in GD [24,25,26,27,28,29,30], though we acknowledge this interpretation goes a step beyond what our data alone can support. *RPH3A* encodes rabphilin-3A, a Rab3-interacting protein involved in vesicle trafficking and membrane fusion; its upregulation may reflect compensatory responses to lysosomal membrane dysfunction, though it has not been specifically studied in GD to our knowledge [31,32]. *SAMSN1* is a signaling adaptor with established roles in macrophage and B-cell activation, and its elevation fits with the broader immune activation signature we observed across both twins [33,34].

The more striking, and more speculative, part of our findings concerns the differences between the two twins. Where they diverged most clearly was in interferon-stimulated gene expression: *ISG15*, *RSAD2*, and *IFI44L* all ran higher in M2 than in M1 and controls, and this pattern was consistent across both RNA sequencing and qRT-PCR. Mean fold changes relative to controls in M2 reached approximately 1.95-fold for *ISG15*, 2.15-fold for *RSAD2*, and 1.72-fold for *IFI44L*, while M1 remained close to or below control levels for all three (*ISG15*: 0.82-fold, *RSAD2*: 0.88-fold, *IFI44L*: 0.93-fold). With only two biological samples per group, no valid inferential test is available, and these results are presented as descriptive fold changes only. The observation we draw attention to is the directional consistency across two independent methods for all three genes—a pattern that, while it cannot be statistically confirmed in a dataset of this size, is coherent enough to warrant investigation in a larger, prospectively designed cohort.

The biological context here is reasonably well developed. Lysosomal dysfunction is now recognized as a trigger for innate immune activation, and glucosylceramide accumulation in GD macrophages has been linked to downstream interferon induced STAT1 signaling [35,36]. Vitner et al. [35] demonstrated induction of the type I interferon response in neurological forms of GD, showing that the 10 most upregulated genes in a severely affected brain region of a GD mouse model were inflammatory genes with a clear interferon signature. Pandey et al. [37] extended this picture to lysosomal storage diseases more broadly, demonstrating that the resulting pro-inflammatory environment drives the generation of pro-inflammatory cytokines, chemokines, growth factors, and multiple components of the complement cascade—collectively contributing to the progressive neurodegeneration characteristic of lysosomal storage diseases with neurological involvement. Melamed et al. [38] reported elevated *IRF7* expression specifically in neurological GD, pointing toward a role for interferon regulatory factors in driving sustained *ISG* activation in more severe disease contexts. Perhaps the most counterintuitive finding in this study was the downregulation of *ISG15*, *RSAD2*, and *IFI44L* in M1—the more severely affected twin—relative to M2. Given that these three genes are canonical markers of IFN pathway activation, and that lysosomal storage burden has been directly linked to innate immune dysregulation and ISG induction in GD [35,38], one might have expected the opposite pattern. A pharmacologically plausible explanation for the attenuated interferon signature in M1 is the corticosteroid premedication that was routinely administered prior to ERT infusions, instituted following an earlier anaphylactic reaction to prevent recurrent hypersensitivity episodes while receiving imiglucerase. Glucocorticoids are well-established suppressors of IFN signaling acting through the glucocorticoid receptor (GR); they impair *ISGF3* transcription complex assembly at interferon-stimulated response elements downstream of JAK-STAT activation, thereby directly attenuating the transcription of interferon-stimulated genes [39]. *ISG15* expression is known to decline following immunosuppressive treatment in autoimmune conditions such as systemic lupus erythematosus SLE [40,41,42]. Previous studies revealed that glucocorticoids suppress type I IFN-β signaling [38,43]. In this light, the reduced expression of interferon associated genes in M1 may not represent a genuinely attenuated interferon response, but rather a pharmacologically imposed silence—raising the unsettling possibility that the true magnitude of IFN-I dysregulation in M1 macrophages is being masked by repeated corticosteroid exposure. This distinction carries meaningful clinical implications: if the IFN-I signature in M1 is suppressed rather than absent, the apparent transcriptional divergence between the twins may substantially underestimate the underlying immunological asymmetry. Resolving this confound from transcriptomic data alone is not feasible, and future studies investigating interferon signatures in ERT-treated patients should prospectively account for corticosteroid premedication history as a critical covariate in their analytical framework.

A second inter-twin difference that emerged from the RNA sequencing data—and one we find harder to interpret—was a modest reduction in snoRNA expression in M2, involving members of the *SNORA* and *SNORD* families: specifically *SNORD32A*, *SNORD21*, *SNORA11*, *SNORA18*, *SNORA25*, and *RNU5E1.* These differences did not reach FDR correction and were not validated by qRT-PCR, so they should be treated with particular caution. snoRNAs are a class of non-coding RNAs classically associated with ribosomal RNA modification and splicing regulation, though their functional scope has expanded considerably in recent years [44,45]. Bratkovič et al. [46] provided a comprehensive account of snoRNA functional diversity, noting that snoRNAs are increasingly implicated in post-transcriptional processes including rRNA acetylation, modulation of splicing patterns, and control of mRNA abundance and translational efficiency [46,47]. Li et al. [48] reviewed snoRNA dysregulation in disease contexts, arguing that abnormal snoRNA expression was associated with a range of conditions including cancer, cardiovascular disease, and neurodegenerative disease, and that the functional consequences of altered snoRNA levels might extend well beyond ribosome biogenesis. One possibility of the suppression of snoRNA clusters in the clinically milder twin (M2) is that it reflects a compensatory post-transcriptional response to chronic lysosomal stress, a kind of adaptive dampening that may partly offset the pro-inflammatory state in GD-affected macrophages and, in doing so, contribute to the milder clinical course observed in this twin. The other possibility, which we cannot dismiss, is that it is simply noise: the sample size is small, and qRT-PCR validation was not performed.

The co-occurrence of snoRNA suppression and interferon-stimulated gene upregulation in M2 is noteworthy, and may reflect the absence of corticosteroid premedication in this twin—leaving the IFN-I pathway unattenuated—as well as the comparatively milder disease burden that characterizes M2’s clinical course. It also raises the possibility that the two twins differ not only in the intensity of their innate immune response, but also in their capacity for post-transcriptional compensation.

We did not perform a dedicated epigenetic analysis. Nevertheless, the transcriptomic differences we observed are also consistent with epigenetic divergence accumulated over decades of distinct life experiences—differing reproductive histories, environmental exposures, and possibly variation in mitochondrial heteroplasmy, which has been shown to influence oxidative stress and gene expression in a tissue-specific manner. Whether differential ERT exposure, epigenetic drift, or a combination of both underlies the inter-twin divergence reported here is a question that will require larger and more deeply phenotyped twin cohorts, combined with longitudinal ERT exposure data, to answer.

A related observation was a more pronounced reduction in ribosomal pseudogene transcript levels in M1 than in M2. Pseudogene-derived transcripts have historically been dismissed as transcriptional noise, but there is now reasonable evidence that some act as competing endogenous RNAs or influence the expression of their parent genes [49,50,51]. Whether what we observed here is functionally meaningful is something we cannot determine from these data, and we mention it only as a direction for future work.

The broader context for all of these findings is the long-standing puzzle of GD phenotypic heterogeneity. Genotype-phenotype correlations in GD are imperfect: the same GBA1 variant can produce a wide range of clinical outcomes, even within families [10,11,52]. Monozygotic twin pairs discordant for GD severity—as documented by Biegstraaten et al. [10]—make this point sharply. Identical genomes do not guarantee identical disease. Epigenetic mechanisms have been increasingly implicated as modifiers of GD severity, including DNA methylation, histone modification, and non-coding RNA regulation [19,53]. The transcriptomic differences we observed between M1 and M2—two individuals with the same *GBA1* genotype—are most parsimoniously explained by epigenetic or post-transcriptional regulatory differences rather than genetic ones. We acknowledge, however, that our study was not designed to test this directly: we did not measure DNA methylation or histone modifications, and transcriptomic data alone cannot distinguish between epigenetic and other non-genetic explanations.

## 5. Limitations

Several limitations of this study deserve direct acknowledgment.

First, and most fundamentally, the sample size is two patients and two controls. This is an inherent constraint of the twin design—discordant monozygotic GD twin pairs are rare—and it means that all findings should be treated as exploratory. It should also be noted that while both patients and one control were female, the second control was male, introducing a degree of sex mismatch that may have influenced gene expression comparisons, particularly given the well-documented sex-related differences in immune gene regulation. The absence of FDR-significant results in the RNA sequencing data is not surprising given the power available; it reflects the sample size, not necessarily the absence of biological signal.

Second, this study relies on in vitro differentiated macrophages. Monocyte-derived macrophages are a well-established model for studying GD [53,54,55], but they may not fully recapitulate the transcriptional state of tissue-resident macrophages in the spleen, liver, or bone marrow—the primary sites of GD pathology. The findings in this model system will need to be validated in more physiologically relevant contexts.

Third, both twins were receiving enzyme replacement therapy at the time of sampling, which may have attenuated some of the GD-associated transcriptional signal [19,20]. We cannot determine from these data whether the expression patterns we observed represent the untreated disease state, a partially treated state, or some combination of the two.

Fourth, we cannot exclude technical variability as a contributor to the inter-twin differences. Although standard normalization procedures were applied, batch effects in a study of this size are difficult to fully control for. The directional concordance between RNA sequencing and qRT-PCR for the six genes we validated reduces this concern somewhat, but does not eliminate it.

Fifth, the qRT-PCR validation covered only three genes from the stress-signaling group (*DDIT4*, *RPH3A*, *SAMSN1*) and three from the interferon group (*ISG15*, *RSAD2*, *IFI44L*).

Sixth, the study design is cross-sectional. We have a single transcriptomic snapshot per participant, which means we cannot assess how expression patterns change over time, in response to treatment, or in relation to clinical progression.

Finally, we acknowledge that the considerable variation in total read counts across the four samples (Patient 1: 301 M; Control 1: 106 M) introduces a potential technical caveat. Although normalization is expected to mitigate the impact of sequencing depth differences, we cannot entirely rule out that coverage heterogeneity contributed to the PC1 separation observed in our principal component analysis, particularly given the small sample size of *n* = 2 per group.

## 6. Conclusions

Using a rare discordant monozygotic twin pair, we found a shared transcriptional pattern in GD macrophages that points toward *DDIT4*-driven mTOR suppression as a possible conserved response to chronic lysosomal stress. On top of this, the more severely affected twin showed higher interferon-stimulated gene expression and somewhat lower snoRNA cluster activity—a pattern that, if it holds up, might reflect differences in post-transcriptional regulation between patients with divergent disease courses. We are careful not to read too much into this; none of these findings are statistically significant, and the sample size does not allow us to be. What we can say is that the observations hang together biologically and are consistent with what others have reported on lysosomal stress and interferon signaling in GD. Whether they represent something real and reproducible is a question for larger studies with proper epigenetic profiling. The twin design gave us an unusually clean comparison—same genome, different disease—and we think that window is worth looking through more carefully.

## Figures and Tables

**Table 1:** Demographic characteristics of twins and healthy individuals.

	Number	Age	Gender	Glucocerebrosidase Enzyme	*GBA* Gene Mutation
Patients					
Monozygotic Twin 1	M1	43	Female	0.25 nmol/h/mg protein (9.4 ± 3.2)	p.Asn409Ser/ p.Asn409Ser
Monozygotic Twin 2	M2	43	Female	0.21 nmol/h/mg protein (9.4 ± 3.2)	p.Asn409Ser/ p.Asn409Ser
Healthy individuals					
Control	C1	43	Male	8 nmol/h/mg protein (9.4 ± 3.2)	-
	C2	45	Female	10.3 nmol/h/mg protein (9.4 ± 3.2)	-

**Table 2:** Clinical and laboratory characteristics of twins.

	Monozygotic Twin 1	Monozygotic Twin 2
Hypertension	+	-
Anaphylaxis to ERT	+	-
Hemoglobin	11.6 g/dL (12–14.6)	12.1 g/dL (12–14.6)
Leucocyte	4.030 × 10 × 10^3^ U/L (4.49–12.68)	5.200 × 10 × 10^3^ U/L (4.49–12.68)
Platelet	174,000 (173,000–290,000)	252,000 (173,000–290,000)
Ferritin	39.7 ng/mL (4.63–204)	45 ng/mL
Chitotriosidase	<1 mol/h/mL	<1 mol/h/mL
Deacylated lysolipid glucosylsphingosine (Lyso GB1)	69.4 nmol/L (<3.7)	43 nmol/L (<3.7)
24 h urine protein	4401 mg/day	206 mg/day
Diastolic septal thickness	1.2 (0.6–1.1) cm indicating mild wall thickening	0.9 (0.6–1.1) cm normal
Diastolic wall thickness	1.1 (0.6–1.1) cm mild increase	0.8 (0.6–1.1) cm normal
Abdominal Ultrasonography	Mild hepatomegaly, craniocaudal (CC) length 14.5 cm, with evidence of hepatic steatosis Mild enlargement of spleen, long axis 13.5 cm	Mild hepatomegaly, craniocaudal (CC) length 12.7 cm, hepatic steatosis Mild enlargement of spleen, long axis 13 cm
Abdominal Magnetic resonance imaging	Mild hepatomegaly, craniocaudal (CC) length 14.5 cm, with evidence of hepatic steatosis Mild enlargement of spleen, long axis 13.5 cm	Mild hepatomegaly, craniocaudal (CC) length 12.7 cm, hepatic steatosis Mild enlargement of spleen, long axis 13 cm
Sacroiliac Magnetic resonance imaging	Normal	Normal
Bone mineral densitometry	Normal	Normal
Electroencephalography	Mild diffuse cerebral dysfunction	Mild diffuse cerebral dysfunction

**Table 3:** Primer sequences used for qRT-PCR analysis.

Gene	Forward Primer	Reverse Primer
*ISG15*	CGCAGATCACCCAGAAGATCG	TTCGTCGCATTTGTCCACCA
*RSAD2*	TGGGTGCTTACACCTGCTG	GAAGTGATAGTTGACGCTGGTT
*IFI44L*	AGCCGTCAGGGATGTACTATAAC	AGGGAATCATTTGGCTCTGTAGA
*DDIT4*	TGAGGATGAACACTTGTGTGC	CCAACTGGCTAGGCATCAGC
*RPH3A*	AGCCGAGATTCAGAGAGCTG	GTTGGCCCGTCTCAAACCT
*SAMSN1*	AGATCCCACAAATGGAAGTGGA	CTCAGAAAGGGCTTTGATGTACT

**Table 4:** The 15 genes exhibiting the most significant variation according to False Discovery Rate (FDR)-adjusted *p*-values.

Gene and Transcript	Patient *	Control *	LogFC ^†^	*p* Value	FDR ^‡^
*RPS26P47* *ENSG00000234354.3*	0.84 (±0.6)	2.95 (±1.4)	−2.47 ▼	4.80 × 10^−4^	8.75 × 10^−1^
*CCL4L2* *ENSG00000276070.5*	1.2 (±0.9)	4.12 (±0.5)	−2.59 ▼	7.85 × 10^−4^	8.75 × 10^−1^
*-* *ENSG00000271581.1*	1.1 (±0.8)	3.71 (±0.3)	−2.55 ▼	8.34 × 10^−4^	8.75 × 10^−1^
*RPS26P58* *ENSG00000225071.1*	0.53 (±0.4)	1.99 (±1.2)	−2.55 ▼	1.02 × 10^−3^	8.75 × 10^−1^
*RPS26P6* *ENSG00000212994.5*	0.64 (±0.4)	2.33 (±1.6)	−2.36 ▼	1.65 × 10^−3^	8.75 × 10^−1^
*-* *ENSG00000136997.21*	0.86 (±0.4)	1.86 (±0.7)	−1.64 ▼	1.69 × 10^−3^	8.75 × 10^−1^
*RPH3A* *ENSG00000089169.15*	1.83 (±1.5)	0.09 (±0.1)	3.74 ▲	1.85 × 10^−3^	8.75 × 10^−1^
*SAMSN1* *ENSG00000155307.19*	2.28 (±1)	0.47 (±0.2)	1.80 ▲	2.01 × 10^−3^	8.75 × 10^−1^
*-* *ENSG00000198019.13*	0.73 (±0.3)	1.66 (±0.2)	−1.76 ▼	2.05 × 10^−3^	8.75 × 10^−1^
*RPS26P8* *ENSG00000204652.6*	0.33 (±0.2)	1.27 (±0.8)	−2.34 ▼	2.11 × 10^−3^	8.75 × 10^−1^
*DDIT4* *ENSG00000168209.6*	2.21 (±0.8)	0.45 (±0.2)	1.82 ▲	2.13 × 10^−3^	8.75 × 10^−1^
*-* *ENSG00000289582.1*	4.9 (±3.1)	12.66 (±0.1)	−2.09 ▼	2.31 × 10^−3^	8.75 × 10^−1^
*-* *ENSG00000196656.7*	2.64 (±2.4)	7.97 (±0.9)	−2.46 ▼	2.45 × 10^−3^	8.75 × 10^−1^
*RPS26P15* *ENSG00000223416.3*	0.39 (±0.2)	1.66 (±1.2)	−2.54 ▼	2.50 × 10^−3^	8.75 × 10^−1^
*GBA1* *ENSG00000177628.16*	0.67 (±0.4)	1.36 (±0.6)	−1.59 ▼	2.85 × 10^−3^	8.75 × 10^−1^

* **TPM Mean (SD)**: Normalized mean read count and standard deviation (Transcripts Per Million); ^†^ **LogFC**: Logarithmic fold change; ^‡^ **FDR:** Adjusted *p*-value (False Discovery Rate); ▼ lower in the patients; ▲ higher in the patients. FDR < 0.05 significant.

**Figure A1:**
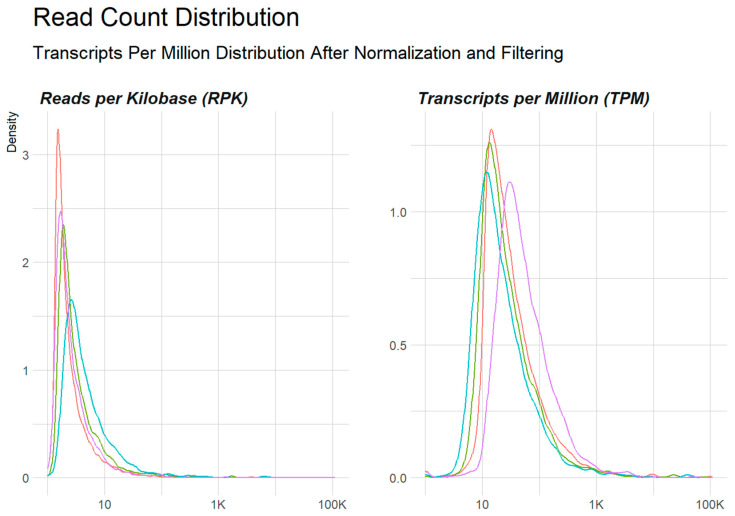
Distribution of all transcripts in raw and normalized data based on TPM (Transcripts per million).

## Data Availability

All data were present in the submission.

## Abbreviations

GD	Gaucher disease
GCase	Glucocerebrosidase
M1	Monozygotic twin 1
M2	Monozygotic twin 2
MRI	Magnetic Resonance İmaging
C	Control
qRT-PCR	Quantitative Real-Time Polymerase Chain Reaction
ERT	Enzyme Replacement Therapy
PCA	Principal component analysis
FDR	False Discovery Rate
*CCL4L2*	C-C Motif Chemokine Ligand 4 Like 2
*SAMSN1*	SH3 domain, and nuclear localization signal 1
*DDIT4*	DNA damage-induced transcription factor 4
mTOR	Mammalian Target Of Rapamycin
*RSAD2*	Radical S-adenosyl methionine domain-containing 2
*IFI44L*	Interferon-Induced Protein 44-Like
IFN	Interferon
JAK	Janus kinase
STAT	Signal Transducer and Activator of Transcription
rRNA	Ribosomal RNA
snoRNA	Small Nucleolar RNA
Lyso Gb1	Deacylated Lysolipid Glucosylsphingosine
*RPH3A*	Rabphilin-3A, Rab3-interacting protein
CC	Craniocaudal

## Appendix A. Supplementary Methods (Expanded Detail)

RNA Isolation and Quality Control

Total RNA was isolated from macrophages using the TRIzol method. RNA quality and quantity were assessed using fluorometric and capillary electrophoresis approaches. RNA concentration was measured with the Qubit 3 Fluorometer (Thermo Fisher Scientific, Waltham, MA, USA; #Q33216), while RNA integrity was evaluated using either the 2100 Bioanalyzer (Agilent Technologies, Santa Clara, CA, USA; #G2939BA) or the 4200 TapeStation (Agilent Technologies, Santa Clara, CA, USA; #G2991BA).

Library Preparation

RNA libraries were prepared using the Illumina Total RNA Prep Kit (Illumina, Inc., San Diego, CA, USA; #20040529). The workflow included ribosomal RNA depletion, random fragmentation of the remaining RNA, synthesis of first- and second-strand cDNA, RNA purification, adapter ligation with index and barcode sequences, and final purification, following the manufacturer’s protocol.

Sequencing

Sequencing was performed on the Illumina NovaSeq 6000 platform, targeting an average of 30 million 150 bp paired-end reads per sample. Library quantification, dilution, and flow cell loading were conducted according to the manufacturer’s guidelines (14).

Quality Control and Preprocessing

Raw sequencing reads were assessed with FASTQC for read counts, base quality, GC content, k-mer distributions, and adapter contamination. Low-quality bases and adapters were trimmed with Trimmomatic v0.39 (15).

Read Alignment and Quantification

Trimmed reads were processed using the Illumina DRAGEN Bio-IT RNA Pipeline, aligned in a splice-aware manner to the Homo sapiens GRCh38.p13 reference genome, with gene, exon, and transcript annotations obtained from GENCODE v40. Transcript abundances were calculated as RPK and normalized to TPM.

## Appendix B. Data Processing

Sample Groups

RNA sequencing was performed on four samples across two experimental groups. Supplementary Table A1 summarizes the sample identifiers, experimental groups, total read counts obtained from sequencing, and the number of reads that are aligned to the reference genome. The “Total” column indicates the total number of reads obtained from each sample; “Aligned” shows the number of reads successfully mapped to the genome; “Alignment Rate” represents the proportion of reads aligned to the genome; “Unaligned” indicates the number of reads that did not map to the genome; and “Q30 Quality Rate” reflects the proportion of reads with a Phred quality score of Q30 or higher.

Filtering and Normalization

It is assumed in the analyses that all samples should exhibit similar ranges and expression distributions. Supplementary Figure A1 illustrates the TPM (Transcripts Per Million) distributions in both raw and normalized data. Each line represents the expression distribution of genes within a sample. Subsequent analyses were conducted using the normalized data. qRT-PCR expression of *DDIT4, RPH3A, SAMSN1* was shown in Table A2 and *ISG15, RSAD2, IFI44L* was shown in Table A3.

**Table A1:** Group information, read counts, and alignment values of the analyzed samples.

	Total	Aligned	Unaligned	Q30 Quality Rate	Alignment Rate
Control					
Control 1	106,369,514	104,052,523	2,316,991	94.73%	97.82%
Control 2	178,575,388	167,349,135	11,226,253	93.43%	93.71%
Patient					
Patient 1	301,446,374	291,846,899	9,599,475	94.36%	96.82%
Patient 2	192,404,672	185,650,207	6,754,465	92.49%	96.49%

**Table A2:** qRT-PCR expression of stress-related genes.

Gene	Controls (Mean ± SD)	M1 (Mean ± SD)	M2 (Mean ± SD)
***DDIT4***	1.00 ± 0.25	2.30 ± 0.35	2.10 ± 0.30
***RPH3A***	1.00 ± 0.20	1.90 ± 0.30	1.70 ± 0.28
***SAMSN1***	1.00 ± 0.22	2.10 ± 0.34	1.90 ± 0.30

Relative expression levels are presented as 2^−ΔΔCt^ values normalized to the housekeeping gene. Data are shown as mean ± SD.

**Table A3:** qRT-PCR expression of interferon-stimulated genes.

Gene	Controls (Mean ± SD)	M1 (Mean ± SD)	M2 (Mean ± SD)
*ISG15*	1.00 ± 0.18	0.82 ± 0.16	1.95 ± 0.35
*RSAD2*	1.00 ± 0.20	0.88 ± 0.17	2.15 ± 0.42
*IFI44L*	1.00 ± 0.16	0.93 ± 0.19	1.72 ± 0.31

Relative expression levels are presented as 2^−ΔΔCt^ values normalized to the housekeeping gene. Data are shown as mean ± SD.

## References

[1] Stirnemann J., Belmatoug N., Camou F., Serratrice C., Froissart R., Caillaud C., Levade T., Astudillo L., Serratrice J., Brassier A. (2017). A Review of Gaucher Disease Pathophysiology, Clinical Presentation and Treatments. Int. J. Mol. Sci..

[2] Mignot C., Gelot A., Bessières B., Daffos F., Voyer M., Menez F., Fallet Bianco C., Odent S., Le Duff D., Loget P. (2003). Perinatal-Lethal Gaucher Disease. Am. J. Med. Genet. A.

[3] Imbalzano G., Ledda C., Romagnolo A., Covolo A., Lopiano L., Artusi C.A. (2024). Neurological Symptoms in Adults with Gaucher Disease: A Systematic Review. J. Neurol..

[4] Camou F., Berger M.G. (2025). Gaucher Disease: State of the Art and Perspectives. J. Intern. Med..

[5] Baldini M., Casirati G., Ulivieri F.M., Cassinerio E., Khouri Chalouhi K., Poggiali E., Borin L., Burghignoli V., Cesana B.M., Cappellini M.D. (2018). Skeletal Involvement in Type 1 Gaucher Disease: Not Just Bone Mineral Density. Blood Cells Mol. Dis..

[6] Mikosch P., Hughes D. (2010). An Overview on Bone Manifestations in Gaucher Disease. Wien. Med. Wochenschr..

[7] Meijon-Ortigueira M.D.M., Solares I., Muñoz-Delgado C., Stanescu S., Morado M., Pascual-Izquierdo C., Blanco L.V., Quintana A.B., Menéndez-Conde C.P., Morales-Conejo M. (2024). Women with Gaucher Disease. Biomedicines.

[8] Goker-Alpan O., Hruska K.S., Orvisky E., Kishnani P.S., Stubblefield B.K., Schiffmann R., Sidransky E. (2005). Divergent Phenotypes in Gaucher Disease Implicate the Role of Modifiers. J. Med. Genet..

[9] Koprivica V., Stone D.L., Park J.K., Callahan M., Frisch A., Cohen I.J., Tayebi N., Sidransky E. (2000). Analysis and Classification of 304 Mutant Alleles in Patients with Type 1 and Type 3 Gaucher Disease. Am. J. Hum. Genet..

[10] Biegstraaten M., van Schaik I.N., Aerts J.M., Langeveld M., Mannens M.M., Bour L.J., Sidransky E., Tayebi N., Fitzgibbon E., Hollak C.E. (2011). A Monozygotic Twin Pair with Highly Discordant Gaucher Phenotypes. Blood Cells Mol. Dis..

[11] Lachmann R.H., Grant I.R., Halsall D., Cox T.M. (2004). Twin Pairs Showing Discordance of Phenotype in Adult Gaucher’s Disease. QJM.

[12] Kopytova A.E., Rychkov G.N., Nikolaev M.A., Baydakova G.V., Cheblokov A.A., Senkevich K.A., Bogdanova D.A., Bolshakova O.I., Miliukhina I.V., Bezrukikh V.A. (2021). Ambroxol Increases Glucocerebrosidase Activity and Restores GCase Translocation in Primary Patient-Derived Macrophages in Gaucher Disease and Parkinsonism. Park. Relat. Disord..

[13] Nikolaev M.A., Kopytova A.E., Baidakova G.V., Emel’yanov A.K., Salogub G.N., Senkevich K.A., Usenko T.S., Gorchakova M.V., Koval’CHuk Y.P., Berkovich O.A. (2019). Human Peripheral Blood Macrophages as a Model for Studying Glucocerebrosidase Dysfunction. Cell Tissue Biol..

[14] Chen L., Zhang Y.H., Wang S., Zhang Y., Huang T., Cai Y.D. (2017). Prediction and analysis of essential genes using the enrichments of gene ontology and KEGG pathways. PLoS ONE.

[15] Bolger A.M., Lohse M., Usadel B. (2014). Trimmomatic: A Flexible Trimmer for Illumina Sequence Data. Bioinformatics.

[16] Robinson M.D., McCarthy D.J., Smyth G.K. (2010). edgeR: A Bioconductor Package for Differential Expression Analysis of Digital Gene Expression Data. Bioinformatics.

[17] Alexa A., Rahnenführer J., Lengauer T. (2006). Improved scoring of functional groups from gene expression data by decorrelating GO graph structure. Bioinformatics.

[18] Yu G., Wang L.-G., Han Y., He Q.-Y. (2012). clusterProfiler: An R Package for Comparing Biological Themes Among Gene Clusters. OMICS.

[19] Dasgupta N., Xu Y.H., Oh S., Sun Y., Jia L., Keddache M., Grabowski G.A. (2013). Gaucher Disease: Transcriptome Analyses Using Microarray or mRNA Sequencing in a Gba1 Mutant Mouse Model Treated with Velaglucerase Alfa or Imiglucerase. PLoS ONE.

[20] Limgala R.P., Jani C., Ioanou C., Alpan O., Goker-Alpan O. (2018). Enzyme Replacement Therapy Reverses B Lymphocyte and Dendritic Cell Dysregulations in Patients with Gaucher Disease. Blood Cells Mol. Dis..

[21] Aflaki E., Moaven N., Borger D.K., Lopez G., Westbroek W., Chae J.J., Marugan J., Patnaik S., Maniwang E., Gonzalez A.N. (2016). Lysosomal Storage and Impaired Autophagy Lead to Inflammasome Activation in Gaucher Macrophages. Aging Cell..

[22] Xu Y.H., Jia L., Quinn B., Zamzow M., Stringer K., Aronow B., Sun Y., Zhang W., Setchell K.D., Grabowski G.A. (2011). Global Gene Expression Profile Progression in Gaucher Disease Mouse Models. BMC Genom..

[23] Kim J.Y., Kwon Y.G., Kim Y.M. (2023). The Stress-Responsive Protein REDD1 and Its Pathophysiological Functions. Exp. Mol. Med..

[24] Kinghorn K.J., Grönke S., Castillo-Quan J.I., Woodling N.S., Li L., Sirka E., Gegg M., Mills K., Hardy J., Bjedov I. (2016). A Drosophila Model of Neuronopathic Gaucher Disease Demonstrates Lysosomal-Autophagic Defects and Altered mTOR Signalling and Is Functionally Rescued by Rapamycin. J. Neurosci..

[25] Sunilkumar S., Dennis M.D. (2024). REDD1 Is a Promising Therapeutic Target to Combat the Development of Diabetes Complications. Diabetes.

[26] Atilano M.L., Hull A.J., Kinghorn K.J. (2024). Autophagic dysregulation triggers innate immune activation in glucocerebrosidase deficiency. Autophagy Rep..

[27] Srikanth M.P., Jones J.W., Kane M., Awad O., Park T.S., Zambidis E.T., Feldman R.A. (2021). Elevated glucosylsphingosine in Gaucher disease induced pluripotent stem cell neurons deregulates lysosomal compartment through mammalian target of rapamycin complex 1. Stem Cells Transl. Med..

[28] Ellisen L.W. (2005). Growth Control under Stress: mTOR Regulation through the REDD1–TSC Pathway. Cell. Cycle.

[29] Pan X., Liu C., Wang X., Zhao M., Zhang Z., Zhang X., Wang C., Song G. (2023). Resveratrol Improves Palmitic Acid-Induced Insulin Resistance via the DDIT_4_/mTOR Pathway in C_2_C_12_ Cells. Mol. Med. Rep..

[30] Li B., Chen R., Chen L., Qiu P., Ai X., Huang E., Huang W., Chen C., Liu C., Lin Z. (2017). Effects of DDIT4 in Methamphetamine-Induced Autophagy and Apoptosis in Dopaminergic Neurons. Mol. Neurobiol..

[31] Maselli R.A., Vázquez J., Schrumpf L., Arredondo J., Lara M., Strober J.B., Pytel P., Wollmann R.L., Ferns M. (2018). Presynaptic congenital myasthenic syndrome with altered synaptic vesicle homeostasis linked to compound heterozygous sequence variants in RPH3A. Mol. Genet. Genom. Med..

[32] Ferrer-Orta C., Pérez-Sánchez M.D., Coronado-Parra T., Silva C., López-Martínez D., Baltanás-Copado J., Gómez-Fernández J.C., Corbalán-García S., Verdaguer N. (2017). Structural characterization of the Rabphilin-3A-SNAP25 interaction. Proc. Natl. Acad. Sci. USA.

[33] Zhu Y.X., Benn S., Li Z.H., Wei E., Masih-Khan E., Trieu Y., Bali M., McGlade C.J., Claudio J.O., Stewart A.K. (2004). The SH3-SAM Adaptor HACS1 Is Up-Regulated in B Cell Activation Signaling Cascades. J. Exp. Med..

[34] Li Y., Li T., Xiao F., Wang L., Liao X., Zhang W., Kang Y. (2025). SAMSN1 Causes Sepsis Immunosuppression by Inducing Macrophages to Express Coinhibitory Molecules That Cause T-Cell Exhaustion via KEAP1–NRF2 Signaling. Chin. Med. J..

[35] Vitner E.B., Farfel-Becker T., Ferreira N.S., Leshkowitz D., Sharma P., Lang K.S., Futerman A.H. (2016). Induction of the Type I Interferon Response in Neurological Forms of Gaucher Disease. J. Neuroinflamm..

[36] Batta G., Soltész L., Kovács T., Bozó T., Mészár Z., Kellermayer M., Szöllősi J., Nagy P. (2018). Alterations in the Properties of the Cell Membrane Due to Glycosphingolipid Accumulation in a Model of Gaucher Disease. Sci. Rep..

[37] Pandey M.K., Burrow T.A., Rani R., Martin L.J., Witte D., Setchell K.D., Mckay M.A., Magnusen A.F., Zhang W., Liou B. (2017). Complement Drives Glucosylceramide Accumulation and Tissue Inflammation in Gaucher Disease. Nature.

[38] Melamed S., Avraham R., Rothbard D.E., Erez N., Israely T., Klausner Z., Futerman A.H., Paran N., Vitner E.B. (2020). Innate Immune Response in Neuronopathic Forms of Gaucher Disease Confers Resistance against Viral-Induced Encephalitis. Acta Neuropathol. Commun..

[39] Flammer J.R., Dobrovolna J., Kennedy M.A., Chinenov Y., Glass C.K., Ivashkiv L.B., Rogatsky I. (2010). The Type I Interferon Signaling Pathway Is a Target for Glucocorticoid Inhibition. Mol. Cell. Biol..

[40] Yuan Y., Ma H., Ye Z., Jing W., Jiang Z. (2018). Interferon-Stimulated Gene 15 Expression in Systemic Lupus Erythematosus: Diagnostic Value and Association with Lymphocytopenia. Z. Rheumatol..

[41] Xiao L., Zhan F., Lin S. (2022). Clinical Values of the Identified Hub Genes in Systemic Lupus Erythematosus. Front. Immunol..

[42] Shen M., Duan C., Xie C., Wang H., Li Z., Li B., Wang T. (2022). Identification of Key Interferon-Stimulated Genes for Indicating the Condition of Patients with Systemic Lupus Erythematosus. Front. Immunol..

[43] Jalkanen J., Pettilä V., Huttunen T., Hollmén M., Jalkanen S. (2020). Glucocorticoids inhibit type I IFN beta signaling and the upregulation of CD73 in human lung. Intensive Care Med..

[44] Dieci G., Conti A., Pagano A., Carnevali D. (2021). Identification of Protein Binding Sites on U3 snoRNA and Pre-rRNA by UV Cross-Linking. RNA Biol..

[45] Gu A.-D., Zhou H., Yu C.-H., Qu L.-H. (2005). A Novel Experimental Approach for Systematic Identification of Box H/ACA snoRNAs from Eukaryotes. Nucleic Acids Res..

[46] Bratkovič T., Božič J., Rogelj B. (2020). Functional Diversity of Small Nucleolar RNAs. Nucleic Acids Res..

[47] Leroy E., Challal D., Pelletier S., Goncalves C., Menant A., Marchand V., Jaszczyszyn Y., van Dijk E., Naquin D., Andreani J. (2025). A Bifunctional snoRNA with Separable Activities in Guiding rRNA 2′-O-Methylation and Scaffolding Gametogenesis Effectors. Nat. Commun..

[48] Li Y., Chen X., Xiao S., Wang H., Li B., Zhang M., Wang K. (2025). Unlocking the Life Code: A Review of SnoRNA Functional Diversity and Disease Relevance. Cell Commun. Signal..

[49] Lu Y., Zhang Y., Hao F., Wang N., Chen Y., Wang J. (2025). Suppression of Pseudogene MT2P1 Transcription Induced by E2F7 Inhibits Hepatocellular Carcinoma Cell Proliferation and Facilitates Apoptosis via Preserving Its Parental Gene. Cancer Biol. Ther..

[50] Poliseno L., Salmena L., Zhang J., Carver B., Haveman W.J., Pandolfi P.P. (2010). A Coding-Independent Function of Gene and Pseudogene mRNAs Regulates Tumour Biology. Nature.

[51] An Y., Furber K.L., Ji S. (2017). Pseudogenes Regulate Parental Gene Expression via ceRNA Network. J. Cell. Mol. Med..

[52] Alvarez M., Delgadillo V., O’Connor J.E., Alfonso P., Giraldo P. (2016). Epigenetic Variability in Gaucher Disease Twins: Different Clinical Manifestations with Identical Genotypes. Blood Cells Mol. Dis..

[53] Panicker L.M., Miller D., Awad O., Bose V., Lun Y., Park T.S., Zambidis E.T., Sgambato J.A., Feldman R.A. (2014). Gaucher iPSC-Derived Macrophages Produce Elevated Levels of Inflammatory Mediators and Serve as a New Platform for Therapeutic Development. Stem Cells.

[54] Aflaki E., Stubblefield B.K., Maniwang E., Lopez G., Moaven N., Goldin E., Marugan J., Patnaik S., Dutra A., Southall N. (2014). Macrophage models of Gaucher disease for evaluating disease pathogenesis and candidate drugs. Sci. Transl. Med..

[55] Elahimanesh M., Ganjali R., Najafi M. (2025). Transcriptomic Signatures in Gaucher Disease Subtypes: A Systems Biology Perspective. Mol. Genet. Metab. Rep..

